# COVID-19 Pediatric Vaccine Hesitancy among Racially Diverse Parents in the United States

**DOI:** 10.3390/vaccines10010031

**Published:** 2021-12-27

**Authors:** Celia B. Fisher, Aaliyah Gray, Isabelle Sheck

**Affiliations:** 1 Center for Ethics Education, Fordham University, Bronx, NY 10458, USA; 2 Department of Psychology, Fordham University, Bronx, NY 10458, USA; agray11@fordham.edu (A.G.); isheck@fordham.edu (I.S.)

**Keywords:** COVID-19, pediatric vaccine hesitancy, racial diversity, children, parents, health disparities

## Abstract

On 29 October 2021, the U.S. FDA authorized the Pfizer-BioNTech COVID-19 (SARS-CoV-2) vaccine for emergency use in children ages 5–11 years. Racial/ethnic minorities have born the greatest burden of pediatric COVID-19 infection and hospitalization. Research indicates high prevalence of parental vaccine hesitancy among the general population, underscoring the urgency of understanding how race/ethnicity may influence parents’ decision to vaccinate their children. Two weeks prior to FDA approval, 400 Hispanic and non-Hispanic Asian, Black, and White parents of children 5–10 years participated in an online survey assessing determinants of COVID-19 pediatric vaccine hesitancy. Compared to 31% Black, 45% Hispanic, and 25% White parents, 62% of Asian parents planned to vaccinate their child. Bivariate and multivariate ordinal logistic regression demonstrated race/ethnicity, parental vaccine status, education, financial security, perceived childhood COVID-19 susceptibility and severity, vaccine safety and efficacy concerns, community support, and FDA and physician recommendations accounted for 70.3% of variance for vaccine hesitancy. Findings underscore the importance of multipronged population targeted approaches to increase pediatric COVID-19 vaccine uptake including integrating health science literacy with safety and efficacy messaging, communication efforts tailored to parents who express unwillingness to vaccinate, and interventions developed in partnership with and delivered through existing trusted community coalitions.

## 1. Introduction

As of 1 October 2021, there have been 1.9 million COVID-19 (SARS-CoV-2) cases among children 5–11 years in the U.S. This has resulted in more than 8300 hospitalizations and at least 94 deaths with infection rates in children less likely to be reported than adult cases [1]. As of October 2021, 30% of children 5–11 years old were hospitalized for COVID-19. Hospitalization rates for specific racial/ethnic groups were 37% non-Hispanic Black, 31% Hispanic, 22% non-Hispanic White and 23% non-Hispanic Asian [1]. Further, Hispanic and non-Hispanic Black children are more likely to be diagnosed with COVID-19-associated multisystem inflammatory syndrome than non-Hispanic Asian and White children [2]. The pandemic has thus exacerbated existing health disparities among these populations [3]. This underscores the urgency of understanding how race/ethnicity may influence parents’ decision to vaccinate their children against COVID-19. As of September 2021, there were no federal data on race/ethnicity for COVID-19 vaccination rates among children 12 years and older. For the seven states reporting such data, non-Hispanic Asian children had higher vaccination rates compared to non-Hispanic Whites, but the rates among White, Hispanic, and Black children were inconsistent [4].

On 29 October 2021, the U.S. Food and Drug Administration (FDA) authorized the Pfizer-BioNTech COVID-19 vaccine for emergency use in children between 5 and 11 years of age [5]. Since the vaccine was approved for children 12–17 years of age, US vaccination rates for this age group have been reported at 42% for the first dose and 32% for series completion [6]. Emerging data on parental intentions to have their 5–11-year-old children vaccinated for COVID-19 following FDA approval have reported similar rates ranging from 44% to 63% in the U.S. [7,8,9,10] and these are consistent with data from across the globe [11,12,13,14,15]. These percentages are troubling since population modeling indicates that vaccinating adolescents and children could reduce overall COVID-related mortality and case load [16]. Since a return to pre-pandemic normality is only achievable with high vaccination rates [17], these rates underscore the importance of identifying predictors of parental pediatric COVID-19 vaccine acceptance and hesitancy for development of effective public health initiatives.

The most consistent predictor of parental COVID-19 vaccine hesitancy in the U.S. is lack of confidence in the safety and effectiveness of the vaccine followed by lack of trust in government and perceptions that children are not susceptible to the disease [8,9,18,19,20,21,22,23]. Demographic variables have also been associated with parental COVID-19 vaccine acceptance. These include lower parental income and education and whether the parent has received the COVID-19 vaccination [9,10,20,22]. A few studies have reported racial/ethnic differences in parental attitudes toward the COVID-19 vaccine for their children. These include higher levels of vaccine hesitancy among Hispanic and non-Hispanic Black parents, lower vaccine safety concerns among non-Hispanic White parents, and higher levels of pediatric COVID-19 vaccination intention among non-Hispanic Asian parents [9,10,18,20,22].

To date, research on racial/ethnic differences in parental COVID-19 vaccine hesitancy has not included a comprehensive analysis of social determinants identified in earlier studies on parental attitudes toward childhood vaccination for longstanding diseases such as influenza, rubella, and HPV [24,25,26,27,28,29,30,31]. These latter studies drew on health beliefs and planned behavior theoretical models to examine relationships between pediatric vaccine hesitancy and disease knowledge, perceived childhood disease susceptibility and symptom severity, vaccine safety and efficacy, trust in government and health care providers, and the influence of community norms. One aim of the current study was to examine the extent to which these factors jointly and independently predict pediatric COVID-19 parental vaccine hesitancy for younger children. The second aim was to assess similarities and differences in the salience of these predictors for Hispanic and non-Hispanic Asian, Black, and White parent populations.

## 2. Methods

Data were collected as part of an online national non-probability survey of 400 English speaking self-identified Hispanic and non-Hispanic Asian, Black, and White female guardians (≥21 years old) of children 5–10 years of age. There were 100 participants for each racial/ethnic group. We selected female guardians (referred to as “parents” in the current study) since they have been found to be significantly less likely than males to plan to vaccinate their school age children [32]. In addition, female guardians are responsible for making 80% of the healthcare decisions for their children [33]. Although the emergency FDA approval is for children 5–11 years of age, we excluded parents with 11-year-olds. Our rationale was that 11-year-olds would soon be eligible for the prior FDA emergency vaccine approval for 12–17-year-olds, of which access to data on population efficacy and safety information for that age group is available [34]. Recruitment and data collection were conducted in October 2021 through Qualtrics XM, a survey aggregator that recruits individuals who sign up to take paid surveys. A total of 1377 females responded to our screener and 977 were screened out due to failure to meet criteria for age, race/ethnicity, or U.S. residency (*n* = 113), respondent’s child was not between 5 and 11 years old (*n* = 199), race/ethnicity quotas were already reached (*n* = 371), participant did not finish the survey (*n* = 153) or pass attention checks (*n* = 75), or the participant declined to consent to research participation (*n* = 66). The protocol was approved by the university institutional review board.

The primary outcome measure was the proportion of parents reporting they planned to give their oldest child between 5 and 10 years old the COVID-19 vaccine once it was approved by the FDA and available for children under 12. Response options included “will definitely not, will probably not, are unsure, probably will, or definitely will” [28]. The survey adapted items from prior scales to assess the following social determinants of parental pediatric COVID-19 vaccine hesitancy: (1) COVID-19 misconceptions (e.g., children have natural immunity and cannot transmit the virus, masks and social distancing are unnecessary in schools, COVID-19 is not any worse than the flu) [35,36]; (2) pediatric COVID-19 susceptibility (e.g., I worry that my child will be exposed to COVID-19 at school this year); (3) severity of the disease for children (e.g., mild symptoms, difficulty breathing, hospitalization, long term health problems) [27,37]; (4) general vaccine hesitancy (e.g., immunizing children is harmful and this fact is covered up, vaccine effectiveness research data are often fabricated); (5) pediatric COVID-19 vaccine safety and efficacy (e.g., the vaccine will significantly reduce my child’s risk of getting sick, the vaccine will be safe for my child [27]; (6) community support for childhood COVID-19 vaccination (e.g., religious leaders; other parents, family members) [26,27]; and (7) trusted sources (e.g., FDA approval, physician recommendation). All items were scored on 6-point Likert-type scales with the exception of true/false items assessing COVID-19 misconceptions. Demographic information included: (1) child’s age, gender, whether the child had been infected with or tested for COVID-19; and (2) parent’s age, education, household income, financial security, COVID-19 vaccination status, and prior testing for or COVID infection. The survey items for this study are available in the supplemental materials for this article.

Descriptive statistics for demographic data, scales, and intent to vaccinate are presented in Table 1 and Table 2. The primary outcome measure was recoded into 3 categories: “no” (definitely or probably not), “unsure”, and “yes” (probably or definitely will). For each scale when appropriate, items were reversed scored for conceptual continuity. Mean scores for each 6-point Likert type response scale were dichotomized into disagree (≤3) and agree (≥3.1); the 9-item cumulative COVID-19 misconception score was dichotomized as low (≤4.5) versus high (≥4.6) level of misconception. Pearson Chi-square tests (χ2) were performed on differences among parent vaccination intentions and for racial/ethnic group differences for each demographic variable and items and scales reflecting beliefs and attitudes. A multivariate logistic regression was performed to evaluate the percent variance and independent influence of demographic and scale scores on intent to vaccinate.

## 3. Results

Overall, 40.75% of parents planned to have their child vaccinated against COVID-19 following FDA approval, 24.75% were unsure, and 34.50% would not vaccinate their child. As illustrated in Table 1, Chi-square tests indicated parents who themselves had been vaccinated for COVID-19, had attended college, reported higher household income, and financial security were significantly more likely to intend to vaccinate their children. There were no differences in vaccination intention for child’s age or gender.

**Figure 1:**
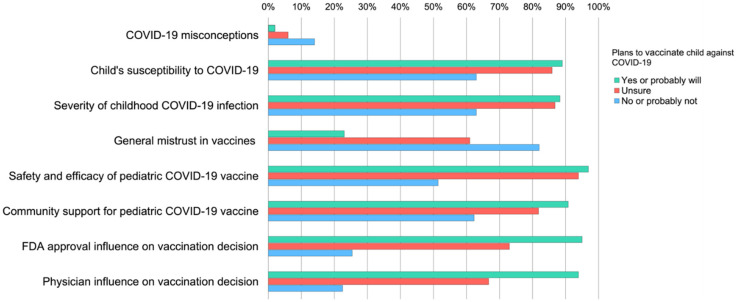
Endorsement of factors influencing pediatric COVID-19 vaccine acceptance and hesitancy by parents’ plans to vaccinate their child against COVID-19. Chi-square tests of association with Bonferonni adjusted *p*-values indicated significant differences among parental plans to vaccinate their child against COVID-19 for all factors, *p* < 0.001.

Figure 1 further illustrates differences in proportion of parents endorsing determinants of vaccine hesitancy by their intent to vaccinate their child against COVID-19 following FDA approval. Chi-square tests indicated parents who did not plan to vaccinate their child had significantly higher levels of COVID-19 misconceptions, perceived children to be less susceptible to infection and believed COVID-19 symptoms were less severe than the parents who were unsure or planned to vaccinate. Compared to the other groups, those who did not plan to vaccinate their children had significantly higher levels of general mistrust for vaccines, lower levels of confidence in the COVID-19 pediatric vaccine safety and efficacy, reported less community support for childhood COVID-19 vaccination and would be less influenced in their vaccination decision by FDA or their doctor’s recommendation. Parents who were unsure had lower levels of vaccine trust and were less influenced by FDA or doctor recommendations than those who planned to vaccinate their children.

Table 2 provides data on demographics, plans to vaccinate, and determinants of vaccine hesitancy for each racial/ethnic group and differences based on Chi-square and analysis of variance tests. Non-Hispanic White parents were significantly older than Hispanic parents. Non-Hispanic Black and White parents reported significantly lower household income compared to Asian and Hispanic parents and a greater proportion of Black parents reported financial insecurity. More Non-Hispanic Asian parents when compared to other racial/ethnic groups reported having received the COVID-19 vaccine themselves. There were no other significant racial/ethnic differences among parental demographic variables.

**Figure 2:**
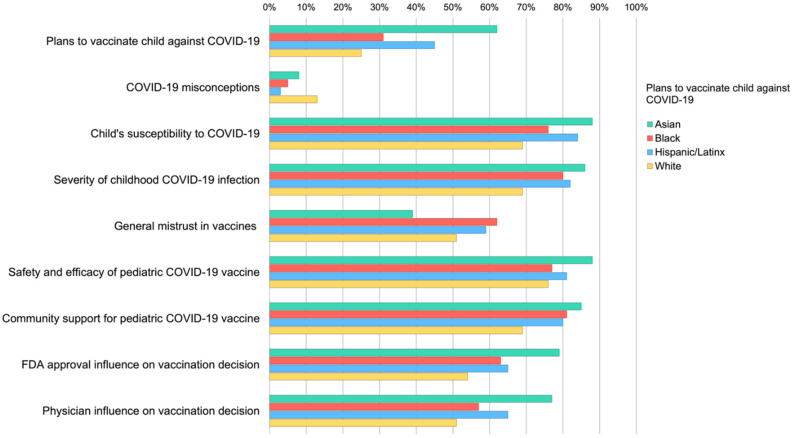
Endorsement of factors influencing parental pediatric COVID-19 vaccine acceptance and hesitancy by race/ethnicity. Chi-square tests of association with Bonferonni adjusted *p*-values indicated that race/ethnicity was significantly associated with plans to vaccinate child against COVID-19 for all variables (range = *p* < 0.05–*p* < 0.001), with the exception of perceived pediatric COVID-19 vaccine safety and efficacy (*p* = 0.13).

As illustrated in Table 2, Chi-square tests indicated significantly higher proportions of non-Hispanic Asian parents planned to vaccinate their child against COVID-19, non-Hispanic Black parents were more likely to report being unsure, and significantly higher proportions of non-Hispanic White parents did not plan to vaccinate their child. Figure 2 further illustrates differences in the proportion of each racial/ethnic group endorsing determinants of vaccine hesitancy. Compared to other racial/ethnic group members, non-Hispanic White parents reported significantly more COVID-19 misconceptions, were less likely to worry their child was susceptible to COVID-19 infection, endorsed less COVID-19 symptom severity if their child became infected and reported overall less community support for vaccinating young children against COVID-19. Non-Hispanic Asian parents were least likely to report general vaccine mistrust and were more likely to endorse positive influences of the FDA and physicians on their decision to vaccinate their child for COVID-19. Across race/ethnicity, the majority of parents indicated some to strong confidence that the COVID-19 vaccine would be safe and effective for their child.

A multivariate ordinal logistic regression that included parental vaccine status, education, financial security, and the hypothesized social determinants as predictors explained 70.3% of the variance (*Nagelkerke* R^2^) in plans to vaccinate one’s child. Parental vaccine status (*p* = 0.004), general trust in vaccines, COVID-19 safety and efficacy concerns, influence of FDA approval, and influence of physician recommendation (*p ≤* 0.001) retained significant independent effects. Race/ethnicity retained a significant independent effect only when comparing Asian and White parents, *p* < 0.001.

## 4. Discussion

In October 2021, 2 weeks prior to the anticipated FDA approval for COVID-19 vaccination of children between the ages of 5 and 11 years, only 41% of a racially and ethnically diverse national sample of female guardians planned to have their young children vaccinated, 25% were unsure, and 34% were unwilling. This percentage is consistent with most prior reports of COVID-19 vaccination rates for children 12–17 years of age and anticipated rates for the emergency use approval for children 5–11 years of age [6,7,8,9,10,11,12,13,14,15]. However, rates significantly differed across racial/ethnic groups with 62% of non-Hispanic Asian parents compared to 31% non-Hispanic Black, 45% Hispanic, and 25% non-Hispanic White planning to vaccinate their child following FDA approval. This study adds to the small but growing literature on parental COVID-19 pediatric vaccine hesitancy by providing an analysis of factors independently and conjointly influencing parents’ decisions to vaccinate their children against COVID-19 infection and how these determinants and decisions vary across U.S. racial/ethnic groups.

Consistent with prior studies on parental hesitancy toward routine pediatric vaccines and emerging data on the current pandemic [8,9,10,18,19,20,21,22,23], bivariate analyses indicated all hypothesized demographic, individual and social determinants were significantly associated with parental intentions to vaccinate their child against COVID-19. Multivariate ordinal logistic regression demonstrated that race/ethnicity (non-Hispanic Asian compared to non-Hispanic White), parental vaccine status, education, financial security, vaccine safety and efficacy concerns, COVID-19 misconceptions and perceived childhood susceptibility to and severity of the disease, community support, and FDA and physician recommendations, accounted for 70.3% of the variance, indicating a goodness-of-fit between the model and parents’ plans to vaccinate their child.

In addition to the contrast in overall beliefs and attitudes between non-Hispanic Asian and White parents, non-Hispanic Asian parents were also less likely to indicate general vaccine mistrust compared to other parents. This may be due in part to the higher proportion of non-Hispanic Asian parents in our sample who themselves received the COVID-19 vaccine. This may indicate a long-standing trust in vaccinations. We also found that although significant in bivariate analyses COVID-19 misconceptions and perceived childhood susceptibility and severity did not sustain independent effects in the logistic regression. One explanation for this finding is that the relationship between vaccine hesitancy and these two determinates was largely driven by non-Hispanic White parents who proportionally reported more disease misconceptions and less susceptibility and severity concerns than other groups. As discussed, below, these parental beliefs may be influenced by vaccine hesitant community norms. Taken together, these findings underscore the importance of understanding racial/ethnic group differences in the design of effective public health strategies.

Our study also adds to existing research by demonstrating how lack of support for pediatric COVID-19 vaccination from other parents, family members, clergy, and others in one’s community is a significant barrier to parental vaccine acceptance. Non-Hispanic White parents in particular, reported significantly less community support (69%) than other groups (80–85%). Moreover, with the exception of non-Hispanic Asian parents, only a little more than half of other parental groups would find their doctor’s recommendation helpful in making a vaccination decision. This pattern of results has implications for public health strategies that have not yet been emphasized in prior research. In some segments of the U.S. population, COVID-19 vaccine hesitancy and uptake interventions may have negligible effects if they focus on clinician-patient communication or expert top-down information sharing messaging. For example, although public service campaigns emphasize speaking to one’s doctor about the vaccine, for most adults and children in the U.S. vaccines have only been available through public testing sites or pharmacies [38]. Thus, even parents with access to a regular pediatrician may not have the opportunity or motivation to contact their physician about this decision. Additionally, national media-based messaging may also be ineffective for populations that traditionally distrust outside experts and or have minimal access to health providers. Targeted approaches for these populations may thus benefit from interventions developed in partnership with and delivered through existing trusted community coalitions such as parent support groups, local political leaders, and religious organizations.

Across racial/ethnic groups, the majority of parents thought the pediatric COVID-19 vaccine would be safe and effective. These findings suggest that public health messaging has been successful in communicating information about the vaccine. Nonetheless, concerns about safety and efficacy were high (48.6%) among parents who would be unwilling to vaccinate their child. This suggests that repeated public health messaging regarding vaccine safety and efficacy alone may be insufficient to increase vaccine acceptance among individuals who may have a general distrust in government or who may be confused by the barrage of information regarding the development and testing of the vaccine. Although there has always been a segment of the public who distrust pediatric vaccines, most parents of young children have trusted in routine vaccines for diseases of childhood which have had a long history of scientific and public acceptance [39,40,41]. In many respects, this is the first time the general public has had a ringside seat to the science of vaccine development and to the process of science itself, including the phased nature of clinical trials, the probabilistic nature of statistically significant results for treatment effectiveness, and the importance of replicability. This suggests that to increase vaccine uptake, public health communications on pediatric COVID-19 vaccine safety and efficacy may need to include strategies to increase health science literacy.

Vaccine hesitancy has been defined as the refusal or delay in the acceptance of a vaccination despite availability of the vaccine or vaccination services [40]. As a consequence, most studies combine into a single group of parents who are unsure and those who express unwillingness to vaccinate their child. Our data suggest, however, that vaccine uptake intervention efforts may benefit from evaluating the unique motivational needs of those who are unsure and those who are unwilling. For example, in our study unwilling parents compared to unsure parents were significantly less worried about children’s susceptibility to the virus (63% vs. 85%) or symptom severity (63% vs. 87%), more distrustful of vaccines in general (82% vs. 60%), less confident in COVID vaccine efficacy and safety (51% vs. 94%), received less community support (64% vs. 83%) and were less likely to see the FDA (25% vs. 73%) or their physician (22% vs. 67%) as trusted sources of information. By contrast, with the exception of distrust in vaccines and in FDA or physician recommendations, endorsements across determinants from unsure parents were not significantly different from those of parents willing to vaccinate their child against COVID-19. As recently suggested for adult vaccine refusers [42], our data suggest that there is a highly resistant group of parents who for multiple reasons are unlikely to be influenced by approaches for addressing vaccine hesitancy that may be successful for unsure parents. Rather, vaccine refusers may benefit from a more comprehensive strategy that addresses the multiple and inter-related barriers to pediatric COVID-19 vaccination that sustains hesitancy among this population.

This study is not without limitations. Our findings are based on cross-sectional data which cannot assess longitudinal causal effects of determinants on parents’ decisions to vaccinate their children. Further, participant recruitment and participation were conducted entirely online through a survey panel aggregator, and consequently, participation was limited to individuals who have access to the internet on web-enabled devices and also who have signed up to complete surveys for compensation. Further, our data suggest that, although we recruited a range of participants from varying SES backgrounds, the majority attended some college and were financially secure. As such, our findings may not adequately represent those from lower socioeconomic backgrounds. Finally, although our study was nationally representative and we found geographic region was not related to parental vaccination intention, we did not assess rural, suburban, or urban differences in parental vaccine hesitancy which may be associated with lack of community support for vaccination and lack of access to and trust in healthcare providers.

## 5. Conclusions

Pediatric vaccine hesitancy among parents is motivated by sociodemographic factors, individual beliefs and attitudes, and social influences that have unique and inter-related effects. Along with the unexpected arrival and spread of COVID-19 infection, the country has witnessed a rapid pace of development of vaccines and treatments for infection accompanied by confusing government and media messaging and increased mistrust in science. This combination of events calls for continued evaluation of the unique and conjoint determinants of parental vaccine hesitancy across distinct groups within the U.S. This is especially true for the development of public health strategies to increase pediatric vaccine uptake across racial/ethnic groups. Early studies on COVID-19 adult vaccine hesitancy have focused on increasing uptake among Hispanic and non-Hispanic Black adults. However, racial/ethnic disparities in vaccination rates continue to narrow, especially for Hispanics [43,44]. Our data suggest that non-Hispanic White populations may be at particular risk of pediatric COVID-19 vaccine refusal. Thus, it is important that the racial/ethnic environment is considered prior to taking public health measures. Future research can benefit from purposive sampling that includes sufficient numbers of distinct racial/ethnic groups to understand unique barriers and facilitators for vaccine acceptance that can inform population tailored interventions. In addition, it may be important for future COVID-19 research and interventions to distinguish among parents who are unsure about vaccinating their children compared with those who may remain highly resistant to public health messaging. Identifying the motivations and effective sources of influence for this group of pediatric vaccine refusers is critical if we are to return to pre-pandemic normality.

## Figures and Tables

**Table 1:** Frequencies and percentages for parent and child characteristics and factors influencing vaccine hesitancy and acceptability by plans to vaccinate child against COVID-19.

	Total Sample *n* = 400	Yes or Probably Will *n* = 163 (40.75%)	Unsure *n* = 99 (24.75%)	No or Probably Not *n* = 138 (34.5%)	*p*-Value
	*n* (%)	*n* (%)	*n* (%)	*n* (%)	
**Parent age**, *M* (*SD*)	35.83 (7.70)	36.70 (6.53)	35.54 (9.50)	35.00 (7.47)	0.15
**Education**					0.02 *
Did not attend college	107 (26.8%)	32 (19.6%)	34 (34.3%)	41 (29.7%)	
Some college or higher	293 (73.3%)	131 (80.4%)	65 (65.7%)	97 (70.3%)	
**Annual household income**					0.004 *
Less than $20,000	156 (39%)	51 (31.3%)	49 (49.5%)	56 (40.6%)	
Between $20,000 and $50,999	178 (44.5%)	75 (46%)	39 (39.4%)	64 (46.4%)	
Between $51,000 and $79,999	49 (12.3%)	27 (16.6%)	5 (5.1%)	17 (12.3%)	
Preferred not to answer	17 (4.3%)	10 (6.1%)	6 (6.1%)	1 (0.7%)	
**Financial security**					0.01 *
Cannot make ends meet	90 (22.5%)	25 (15.3%)	30 (30.3%)	35 (25.4%)	
Have just enough or comfortable	310 (77.5%)	138 (84.7%)	69 (69.7%)	103 (74.6%)	
**Region of residence**					0.17
Northeast	66 (16.5%)	29 (17.8%)	15 (15.2%)	22 (15.9%)	
Midwest	132 (33%)	53 (32.5%)	41 (41.4%)	38 (27.5%)	
South	110 (27.5%)	40 (24.5%)	29 (29.3%)	41 (29.7%)	
West	92 (23%)	41 (25.2%)	14 (14.1%)	37 (26.8%)	
**Parent vaccination status**					<0.001 *
No	173 (43.3%)	19 (11.7%)	51 (51%)	103 (74.6%)	
Yes	227 (56.8%)	144 (88.3%)	48 (48.5%)	35 (25.4%)	
**Parent previously tested for COVID-19**					0.71
No	191 (47.8%)	81 (49.7%)	44 (44.4%)	66 (47.8%)	
Yes	209 (52.3%)	82 (50.3%)	55 (55.6%)	72 (52.2%)	
**Child’s age,***M* (*SD*)	7.66 (1.70)	7.94 (1.68)	7.49 (1.62)	7.46 (1.74)	0.12
5 to 7 years old	194 (48.5%)	69 (42.3%)	52 (52.5%)	73 (52.9%)	
8 to 10 years old	206 (51.5%)	94 (57.7%)	47 (47.5%)	64 (47.1%)	
**Child’s gender**					0.16
Male	214 (53.5)	77 (47.2%)	61 (61.6%)	76 (55.1%)	
Female	185 (46.3)	85 (52.1%)	38 (38.4%)	62 (44.9%)	
Gender nonbinary	1 (0.3)	1 (0.6%)	0%	0%	
**Child previously tested for COVID-19**					0.81
No	200 (50%)	80 (49.1%)	49 (49.5%)	71 (51.4%)	
Yes	196 (49%)	81 (49.7%)	50 (50.5%)	65 (47.1%)	
I don’t know	4 (1%)	2 (1.2%)	0%	2 (1.4%)	
**Child previously had COVID-19**					0.34
No	437 (86.8%)	144 (88.3%)	88 (88.9%)	115 (83.3%)	
Yes	0%	0%	0%	0%	
I don’t know	54 (13.3%)	19 (11.7%)	11 (11.1%)	23 (16.7%)	
**COVID-19 misconceptions**					<0.001 *
*M* (*SD*)	1.47 (1.65)	.88 (1.23)	1.30 (1.51)	2.28 (1.85)	
More COVID-19 misconceptions	29 (7.2%)	3 (1.8%)	6 (6.1%)	20 (14.5%)	
Less COVID-19 misconceptions	371 (92.8%)	160 (98.2%)	93 (93.9%)	118 (85.5%)	
**Child’s susceptibility to COVID-19**					<0.001 *
*M* (*SD*)	4.06 (1.33)	4.39 (1.06)	4.37 (1.22)	3.45 (1.49)	
Greater perceived susceptibility	317 (79.3%)	145 (89%)	85 (85.9%)	87 (63%)	
Less perceived susceptibility	83 (20.8%)	18 (11%)	14 (14.1%)	51 (37%)	
**Severity of childhood COVID-19 infection**					<0.001 *
*M* (*SD*)	3.87 (1.06)	4.17 (.96)	4.03 (.96)	3.41 (1.09)	
Greater perceived severity	317 (79.3%)	144 (88.3%)	86 (86.9%)	87 (63%)	
Less perceived severity	83 (20.8%)	19 (11.7%)	13 (13.1%)	51 (37%)	
**General mistrust in vaccines**					<0.001 *
*M* (*SD*)	3.17 (1.24)	2.41 (1.03)	3.25 (.89)	4.01 (1.14)	
Greater mistrust in vaccines	211 (52.8%)	38 (23.3%)	60 (60.6%)	113 (81.9%)	
Less mistrust in vaccines	189 (47.3%)	125 (76.7%)	39 (39.4%)	25 (18.1%)	
**Safety and efficacy of pediatric COVID-19 vaccine**					<0.001 *
*M* (*SD*)	3.96 (1.19)	4.79 (.89)	3.97 (.80)	2.98 (.95)	
Greater perceived safety and efficacy	322 (80.5%)	158 (96.6%)	93 (93.9%)	71 (51.4%)	
Less perceived safety and efficacy	78 (19.5%)	5 (3.1%)	6 (6.1%)	67 (48.6%)	
**Community support for pediatric COVID-19 vaccination**					<0.001 *
*M* (*SD*)	3.26 (0.94)	3.68 (0.86)	3.23 (0.81)	2.78 (0.89)	
Greater perceived community support	315 (78.8%)	148 (90.8%)	81 (81.8%)	86 (62.3%)	
Less perceived community support	85 (21.3%)	15 (9.2%)	18 (18.2%)	52 (37.7%)	
**FDA approval influence on vaccination decision**					<0.001 *
*M* (*SD*)	3.99 (1.70)	5.20 (.99)	4.04 (1.17)	2.52 (1.52)	
Greater influence of FDA approval	261 (65.3%)	154 (94.5%)	72 (72.7%)	35 (25.4%)	
Less influence of FDA approval	139 (34.8%)	9 (5.5%)	27 (27.3%)	103 (74.6%)	
**Physician influence on vaccination decision**					<0.001 *
*M* (*SD*)	3.88 (1.69)	5.06 (.99)	3.99 (1.30)	2.39 (1.43)	
Greater influence of physician recommendation	250 (62.5%)	153 (93.6%)	66 (66.7%)	31 (22.5%)	
Less influence of physician recommendation	150 (37.5%)	10 (6.1%)	33 (33.3%)	107 (77.5%)	

Note. Statistical tests: Analysis of Variance (ANOVA) for parent age; Chi-square tests of independence for all other variables. * indicates significance, *p* < 0.05, 0.01, or 0.001.

**Table 2:** Frequencies and percentages for parent and child characteristics, plans to vaccinate child against COVID-19, and factors influencing COVID-19 vaccine hesitancy and acceptability by race/ethnicity.

	Total Sample (*n* = 400)	Non-Hispanic Asian (*n =* 100)	Non-Hispanic Black (*n* = 100)	Hispanic/ Latinx (*n* = 100)	Non-Hispanic White (*n* = 100)	*p*-Value
	*n* (%)	*n* (%)	*n* (%)	*n* (%)	*n* (%)	
**Parent age**, *M (SD)*	35.83 (7.70)	36.13 (5.74)	35.90 (8.02)	33.91 (7.24)	37.37 (9.10)	0.01 *
**Education**						0.25
Did not attend college	107 (26.8%)	19%	30%	29%	29%	
Some college or higher	293 (73.3%)	81%	70%	71%	71%	
**Annual household income**						<0.001 *
Less than $20,000	156 (39%)	31%	52%	31%	42%	
Between $20,000 and $50,999	178 (44.5%)	43%	41%	54%	40%	
Between $51,000 and $79,999	49 (12.3%)	15%	4%	14%	16%	
Preferred not to answer	17 (4.3%)	11%	3%	1%	2%	
**Financial security**						0.04
Cannot make ends meet	90 (22.5%)	15%	32%	21%	22%	
Have just enough or comfortable	310 (77.5%)	85%	68%	79%	78%	
**Region of residence**						0.23
Northeast	66 (16.5%)	14%	13%	15%	24%	
Midwest	132 (33%)	34%	34%	36%	28%	
South	110 (27.5%)	23%	35%	25%	27%	
West	92 (23%)	29%	18%	24%	21%	
**Parent vaccination status**						<0.001 *
No	173 (43.3%)	23%	56%	42%	52%	
Yes	227 (56.8%)	77%	44%	58%	48%	
**Parent previously tested for COVID-19**						0.15
No	191 (47.8%)	53%	46%	39%	53%	
Yes	209 (52.3%)	47%	54%	61%	47%	
**Child’s age,***M* (*SD*)	7.66 (1.70)	7.88 (1.59)	7.81 (1.72)	7.81 (1.73)	7.14 (1.67)	<0.001 *
5 to 7 years old	194 (48.5%)	44%	43%	40%	67%	
8 to 10 years old	206 (51.5%)	56%	57%	60%	33%	
**Child’s gender**						0.16
Male	214 (53.5)	44%	54%	62%	54%	
Female	185 (46.3)	55%	46%	38%	46%	
Gender nonbinary	1 (0.3)	1%	0%	0%	0%	
**Child previously tested for COVID-19**						0.44
No	200 (50%)	58%	46%	45%	51%	
Yes	196 (49%)	42%	52%	54%	48%	
I don’t know	4 (1%)	0%	2%	1%	1%	
**Child previously had COVID-19**						0.03 *
No	437 (86.8%)	94%	89%	81%	83%	
Yes	0%	0%	0%	0%	0%	
I don’t know	54 (13.3%)	6%	11%	18%	17%	
**Plans to vaccinate child against COVID-19**						<0.001 *
No or probably not	138 (34.5%)	22%	34%	32%	50%	
Unsure	99 (24.8%)	16%	35%	23%	25%	
Yes or probably will	163 (40.8%)	62%	31%	45%	25%	
**COVID-19 misconceptions**						0.04 *
*M* (*SD*)	1.47 (1.65)	1.41 (1.78)	1.29 (1.48)	1.40 (1.40)	1.77 (1.88)	
More COVID-19 misconceptions	29 (7.2%)	8%	5%	3%	13%	
Less COVID-19 misconceptions	371 (92.8%)	92%	95%	97%	87%	
**Child’s susceptibility to COVID-19**						0.01 *
*M* (*SD*)	4.06 (1.33)	4.37 (1.15)	4.10 (1.31)	4.25 (1.31)	3.51 (1.41)	
Greater perceived susceptibility	317 (79.3%)	88%	76%	84%	69%	
Less perceived susceptibility	83 (20.8%)	12%	24%	16%	31%	
**Severity of childhood COVID-19 infection**						0.02 *
*M* (*SD*)	3.87 (1.06)	4.05 (.93)	3.86 (1.19)	4.07 (1.01)	3.51 (1.01)	
Greater perceived severity	317 (79.3%)	86%	80%	82%	69%	
Less perceived severity	83 (20.8%)	14%	20%	18%	31%	
**General vaccine mistrust**						0.01 *
*M* (*SD*)	3.17 (1.24)	2.81 (1.20)	3.38 (1.13)	3.32 (1.34)	3.18 (1.23)	
Greater vaccine mistrust	211 (52.8%)	39%	62%	59%	51%	
Less vaccine mistrust	189 (47.3%)	61%	38%	41%	49%	
**Safety and efficacy of pediatric COVID-19 vaccine**						0.13
*M* (*SD*)	3.96 (1.19)	4.27 (1.05)	3.82 (1.18)	4.00 (1.25)	3.76 (1.200)	
Greater perceived safety and efficacy	322 (80.5%)	88%	77%	81%	76%	
Less perceived safety and efficacy	78 (19.5%)	12%	23%	19%	24%	
**Community support for pediatric COVID-19 vaccination**						0.04 *
*M* (*SD*)	3.26 (0.94)	3.50 (0.99)	3.22 (0.83)	3.33 (0.96)	2.99 (0.93)	
Greater perceived community support	315 (78.8%)	85%	81%	80%	69%	
Less perceived community support	85 (21.3%)	15%	19%	20%	31%	
**FDA approval influence on vaccination decision**						0.003 *
*M* (*SD*)	3.99 (1.70)	4.58 (1.49)	3.89 (1.66)	3.99 (1.68)	3.50 (1.79)	
Greater influence of FDA approval	261 (65.3%)	79%	63%	65%	54%	
Less influence of FDA approval	139 (34.8%)	21%	37%	35%	46%	
**Physician influence on vaccination decision**						0.001 *
*M* (*SD*)	3.88 (1.69)	4.42 (1.58)	3.62 (1.66)	4.00 (1.65)	3.46 (1.72)	
Greater influence of physician recommendation	250 (62.5%)	77%	57%	65%	51%	
Less influence of physician recommendation	150 (37.5%)	23%	43%	35%	49%	

Note. Statistical tests: Analysis of Variance (ANOVA) for parent age; Chi-square tests of independence for all other variables. * indicates significance above *p* < 0.05.

## Data Availability

Data supporting the reported results is provided on the Fordham University download data portal at https://www.fordham.edu/info/24019/center_for_ethics_education_research/12457/pediatric_covid-19_parental_hesitancy_and_racialethnic_diversity_supplementary_materials, accessed on 24 December 2021.

## Supplementary Materials

The following are available online at https://www.mdpi.com/article/10.3390/vaccines10010031/s1. COVID-19 Pediatric Vaccine Hesitancy among Racially Diverse Parents in the United States Pediatric COVID-19 Parental Hesitancy (PCPH)—Survey items.
