# Supplementary material for: COVID-19 Pediatric Vaccine Hesitancy among Racially Diverse Parents in the United States
Source: Vaccines (Basel). 2021 Dec 27;10(1):31. doi: 10.3390/vaccines10010031 (PMC8778198; doi:10.3390/vaccines10010031)
Supplement: Supplementary file 1 [file vaccines-10-00031-s001.zip › vaccines-1506903-supplementary.pdf]

vaccines

COVID-19 Pediatric Vaccine Hesitancy among Racially Diverse Parents in the United States

Celia B. Fisher, Aaliyah Gray and Isabelle Sheck

COVID-19 Pediatric Vaccine Hesitancy among Racially Diverse Parents in the United States

Pediatric COVID-19 Parental Hesitancy (PCPH) – **Survey items**

Survey Items	Response options
**Parent race**	
Of these commonly used categories, which do you consider to be your primary race/ethnicity?	• American Indian/Native American or Alaska Native Asian<br>• Black, African American, West Indian, Caribbean<br>• Hispanic or Latinx<br>• Native Hawaiian or Other Pacific Islander<br>• White<br>• Other Race
**Parent age**	
How old are you?	Open text response
**Education**	
What is the highest grade or level of school that you have completed?	• 8th grade or less<br>• Partial high school<br>• High school graduate<br>• Partial college (at least one year)<br>• Undergraduate college degree<br>• Graduate degree<br>• I don't know
**Annual household income**	
What is your annual household income (your income and the income of everyone who lives with you and contributes to the household budget) before taxes?	• Less than $5000<br>• $5000 – $19,999<br>• $20,000 – $30,999<br>• $31,000 – $50,999<br>• $51,000 – $79,999<br>• $80,000 – $100,000<br>• More than $100,000<br>• I do not wish to answer
**Financial security**	
How would you describe your current financial situation?	• I cannot make ends meet

	• I have just enough<br>• I am comfortable
**Region of residence**	
Name the state in which you are completing this survey<br>(recoded into Northeast, Midwest, South, West)	Dropdown of states in the U.S.
**Parent vaccination status**	
Have you received a COVID-19 vaccine?	Yes/No
**Parent previously tested for COVID-19**	
Have you ever had COVID-19? (Recoded not yes or no)	• No, but I have not been tested No, I received test results that were negative<br>• Yes, but I have not been tested Yes, I received test results that were positive<br>• I do not wish to answer
**Child age**	
What is the age of your child?	• 5<br>• 6<br>• 7<br>• 8<br>• 9<br>• 10
**Child gender**	
What is the gender of your child?	• Male<br>• Female<br>• Transgender<br>• Gender non-binary
**Child previously tested for COVID-19**	
Has your child ever been tested for COVID 19?	• Yes<br>• No<br>• I don't know
**Child previously had COVID-19**	
Has your child ever had COVID 19?	• Yes<br>• No<br>• I don't know
**COVID-19 misconceptions** (9 items)	
• Some of the symptoms of COVID-19 in children and adults are fever, fatigue, and dry cough.	True/False

• Children and adults who are infected with COVID-19 cannot transmit the virus to others if they do not have symptoms.<br>• The COVID-19 virus spreads via droplets from infected individuals.<br>• Masks are effective at preventing COVID-19 infections in schools.<br>• It is not necessary for children to wear masks and practice social distancing because they are not likely to get sick.<br>• It is not necessary for children to get vaccinated against COVID-19 because they are naturally immune to the virus.<br>• The health risks from COVID-19 have been exaggerated.<br>• COVID-19 is a hoax.<br>• COVID-19 isn't any worse than the flu.	
**Child's susceptibility to COVID-19** (5 items)	
• I worry that my child will be exposed to COVID-19 at school this year.<br>• I worry that my child will be exposed to COVID-19 indoors playing with friends.<br>• I worry that my child will be exposed to COVID-19 playing outside with friends.<br>• I worry that my child will be exposed to COVID-19 at a close friend or family member's house.<br>• I worry that my child will be exposed to COVID-19 by me or someone else living in my home.	6-point Likert scale (Strongly disagree – Strongly agree)
**Severity of childhood COVID-19 infection** (4 items)	
• If my child became infected with COVID-19 it would probably only cause mild symptoms (e.g., cough, fever, body aches) that they would easily recover from.<br>• If my child became infected with COVID-19 it could cause serious symptoms (e.g., difficulty breathing).<br>• If my child became infected with COVID-19 there is a strong possibility that they would need to be hospitalized.<br>• If my child became infected with COVID-19 they could have long term health problems even after they recover.	6-point Likert scale (Strongly disagree – Strongly agree)
**General mistrust in vaccines** (10 items)	
• Vaccines are a good way to protect public health.<br>• Vaccinating children is a good idea.<br>• I personally do not like the idea of vaccines<br>• Doctors give out too many vaccines.<br>• Immunizing children is harmful and this fact is covered up.<br>• Pharmaceutical companies cover up the dangers of vaccines.<br>• People are deceived about whether the vaccine actually works.<br>• People are deceived about vaccine safety.<br>• Vaccine effectiveness research data is often fabricated.<br>• The government is trying to cover up the link between vaccines and autism.	6-point Likert scale (Strongly disagree – Strongly agree)
**Safety and efficacy of pediatric COVID-19 vaccine** (7 items)	

vaccines

MDPI

• The vaccine will be effective.<br>• The vaccine will significantly reduce my child's risk of getting sick.<br>• The vaccine will be safe for my child.<br>• The vaccine will interfere with my child's current medication.<br>• Giving my child the vaccine will be against my religious beliefs.<br>• Giving my child the vaccine will be like performing an experiment on my child.<br>• The vaccine will lead to long-term health problems for my child.	6-point Likert scale (Strongly disagree – Strongly agree)
**Community support for pediatric COVID-19 vaccination** (7 items)	
• Religious leaders<br>• Political leaders<br>• Doctors and healthcare providers<br>• Other parents in my community<br>• My friends<br>• My child's teachers<br>• My family	6-point Likert scale (Strongly unsupportive – Strongly supportive)
**FDA approval influence on vaccination decision**	
The likelihood that I will have my 5 - 10 year old child receive the COVID-19 vaccine will increase if the FDA approves it as safe for children under 12.	6-point Likert scale (Strongly disagree – Strongly agree)
**Physician influence on vaccination decision**	
The likelihood that I will have my 5 - 10 year old child receive the COVID-19 vaccine will increase if my doctor recommends it for my child.	6-point Likert scale (Strongly disagree – Strongly agree)
**Plans to vaccinate child against COVID-19**	
Please indicate which statement is closest to where you are now in your plans to give your child who is between the ages of 5 - 10 years old a COVID-19 vaccine once it is approved by the FDA for children under 12.	• I will definitely not have my child get the vaccine<br>• I will probably not have my child get the vaccine<br>• I am thinking about getting my child the vaccine but I am unsure<br>• I will probably have my child get the vaccine<br>• I will definitely have my child get the vaccine
